# Diarrhea, Pneumonia, and Infectious Disease Mortality in Children Aged 5 to 14 Years in India

**DOI:** 10.1371/journal.pone.0020119

**Published:** 2011-05-24

**Authors:** Shaun K. Morris, Diego G. Bassani, Shally Awasthi, Rajesh Kumar, Anita Shet, Wilson Suraweera, Prabhat Jha, for the MDS Collaborators

**Affiliations:** 1 Centre for Global Health Research, Li Ka Shing Knowledge Institute, St Michael's Hospital, Dalla Lana School of Public Health, University of Toronto, Toronto, Ontario, Canada; 2 Division of Infectious Diseases, Hospital for Sick Children, University of Toronto, Toronto, Ontario, Canada; 3 Department of Pediatrics, King George's Medical University, Lucknow, Uttar Pradesh, India; 4 School of Public Health, Post Graduate Institute of Medical Education, Chandigarh, India; 5 Department of Pediatrics, St. John's National Academy of Health Sciences, Bangalore, India; Kenya Medical Research Institute - Wellcome Trust Research Programme, Kenya

## Abstract

**Background:**

Little is known about the causes of death in children in India after age five years. The objective of this study is to provide the first ever direct national and sub-national estimates of infectious disease mortality in Indian children aged 5 to 14 years.

**Methods:**

A verbal autopsy based assessment of 3 855 deaths is children aged 5 to 14 years from a nationally representative survey of deaths occurring in 2001–03 in 1·1 million homes in India.

**Results:**

Infectious diseases accounted for 58% of all deaths among children aged 5 to 14 years. About 18% of deaths were due to diarrheal diseases, 10% due to pneumonia, 8% due to central nervous system infections, 4% due to measles, and 12% due to other infectious diseases. Nationally, in 2005 about 59 000 and 34 000 children aged 5 to 14 years died from diarrheal diseases and pneumonia, corresponding to mortality of 24·1 and 13·9 per 100 000 respectively. Mortality was nearly 50% higher in girls than in boys for both diarrheal diseases and pneumonia.

**Conclusions:**

Approximately 60% of all deaths in this age group are due to infectious diseases and nearly half of these deaths are due to diarrheal diseases and pneumonia. Mortality in this age group from infectious diseases, and diarrhea in particular, is much higher than previously estimated.

## Introduction

Diarrheal diseases, pneumonia, and other infectious diseases are leading causes of death among children younger than five years in low and middle income countries and also in India [1], [2]. However, little is known about the causes of death in children after age five years. The Global Burden of Disease and Risk Factors (GBD) estimates that in 2004 there were approximately 69 000 deaths from pneumonia and 1000 deaths from diarrheal diseases among children aged 5–14 years in India, accounting for approximately 20% of all deaths at these ages [3]. However, medical certification of deaths in India and other south Asian countries is uncommon. Thus, estimates of mortality in this age group are largely derived from models based on estimates from countries where vital registration data is available and older, non-representative studies of causes of death in India.

The objective of this study is to provide the first ever direct national and sub-national estimates of deaths from the major infectious causes among Indian children ages 5 to 14 years, with a focus on diarrheal diseases and pneumonia.

## Methods

### Ethics Statement

SRS enrolment is on a voluntary basis, and its confidentiality and consent procedures are defined as part of the Registration of Births and Deaths Act, 1969. Oral consent was obtained in the first SRS sample frame. The new SRS sample obtains written consent at the baseline. Families are free to withdraw from the study, but the compliance is close to 100%. The study poses no or minimal risks to enrolled subjects. All personal identifiers present in the raw data are anonymized before analysis. The study has been approved by the review boards of the Post-Graduate Institute of Medical Education and Research, the Indian Council of Medical Research, the Indian Health Ministry's Screening Committee, and by St. Michael's Hospital in Toronto, Canada.

### Study Methods

The Million Death Study (MDS) [4] is conducted within the Registrar General of India's (RGI) nationally representative Sampling Registration System (SRS). Since 1971, the SRS has served as a large, routine, demographic survey to collect information on fertility and mortality in India. The SRS sampling frame used for this study surveyed all deaths occurring among a sample of 6·3 million people in 1·1 million nationally representative Indian households between 2001 and 2003 [5]. An average of 150 households were selected from each of 6 671 sampling units chosen randomly to be representative at the state, urban, and rural level for all 35 states and union territories of India. Within the SRS, selected households were monitored for vital events on a monthly basis by a part-time enumerator and every 6 months by a full-time surveyor from the RGI. For all deaths identified in these households, a standard verbal autopsy (VA) questionnaire, termed RHIME (Routine, Reliable, Representative and Re-sampled Household Investigation of Mortality with Medical Evaluation) was administered. The VA questionnaire used both an open-ended narrative and close-ended questions [6]. Trained Registrar General of India surveyors administered the questionnaire. Two physicians independently reviewed each completed RHIME and assigned a single cause of death using the International Classification of Disease-10 (ICD-10) [7].

The classification system of ICD-10 codes into categories of cause of death in this study is the same as that used in 1 to 59 month old children in our recently published study of under 5 mortality in India (Table S1) [2]. If the two physicians disagreed on the cause of death, an attempt was made to reconcile to a common ICD-10 code. If disagreement persisted, a third senior physician adjudicated the final cause of death.

Total population and deaths among children aged 5 to 14 years by gender and at the state level and by rural and urban areas were proportionally corrected to reflect the UN Population Division estimates for India in 2005 [8]. The estimated number of deaths was calculated by applying the regional, gender, and age (age groups 5 to 9 and 10 to 14 years) specific proportion of deaths from each cause to the estimated total number of deaths in each region. These methods are described in detail elsewhere [4], [9]. All proportions were weighted to account for the survey sampling design. Mortality rates per 100 000 population were calculated for each gender and region [2].

The major source of uncertainty in the regional and gender variation in cause-specific mortality rates in this study is the cause of death proportion. We have calculated 99% confidence intervals for all estimates of proportions of causes of death based on the observed number of deaths in the study and the survey design and sampling. The UN annual estimates of total number of livebirths and deaths by country are widely accepted and the corresponding uncertainty bounds are also made available by the UN Population Division.

Analyses of the seasonality of diarrheal and pneumonia deaths used the monthly weighted average of deaths over three years (2001–03). There were more deaths than expected in January 2001, the first month of data collection and this may be an artifact of the data collection procedure. We thus used an average of the number of deaths in January 2002 and 2003 for the January seasonal estimate. All statistical analyses were performed using Stata/SE version 10.1 [10].

## Results

There were a total of 3 855 deaths in children aged 5 to 14 years from 2001 to 2003 in the study sample. Based on the total number of deaths by gender, the overall all cause mortality rate for boys and girls aged 5 to 14 years was 130 and 148 per 100,000 respectively. Table 1 shows the top causes of death at these ages. In boys aged 5 to 14 years, diarrhea was the cause of 12·0% (99% CI; 8·7 to 16·3) and pneumonia was the cause of 4·3% (99% CI; 2·5 to 7·3) of all deaths. In girls aged 5 to 9 years, diarrhea was the cause of 17·3% (99%CI; 13·3 to 22·3) and pneumonia the cause of 6·9% (99% CI; 4·5 to 10·3) of all deaths. In the 10 to 14 year old age group, diarrhea was the cause of death of 17·0% (99% CI; 14·2 to 20·2) of boys and 20·6% (99% CI; 17·7 to 23·9) of girls whereas pneumonia was the cause of 10·9% (99% CI; 8·6 to 13·7) and 13·6% (99% CI 11·1 to 16·5) of boy and girls deaths respectively. The distribution of ICD-10 codes for the diarrheal and pneumonia deaths are shown in Table S2. Malaria (8·3%, 99% CI; 6·7 to 9·0), central nervous system infections (6·1%, 99% CI; 5·1 to 7·4), and measles (4·5%, 99% CI; 3·6 to 5·5) constituted the remaining top five infectious diseases causes of death in all children aged 5 to 14 years. The remainder of the deaths in children aged 5 to 14 years were due to injury (21·6%, 99% CI; 19·0 to 24·3), non-communicable diseases (20·6%, 99% CI; 18·8 to 22·5), and other infectious diseases (11·7%, 99% CI; 10·3 to 13·2). Overall, 86% of deaths occurred in rural areas (range from 82% for non communicable diseases to 94% for malaria) and only 16% occurred in a health facility. The most common symptoms preceding death from the major infectious diseases are shown in Table 2. The remainder of this paper will focus on diarrheal and pneumonia mortality since a complete analysis of malaria mortality in India (including children) is published elsewhere [11].

**Table 1:** Causes of Death in Children 5 to 14 Years old and Comparison to Global Burden of Disease Estimates.

		MDS Proportions				Estimated Deaths (thousands)				
Cause of Death	Study Deaths 2001–30	Total	Rural Area	Death in Health Facility	Boys	99% CI	Girls	99% CI	Total	99% CI
Diarrheal Diseases	675	0·18	0·86	0·12	25·1	(21·9–29·8)	34·2	(30·0–38·9)	59·3	(51·9–68·7)
Pneumonia	387	0·10	0·87	0·10	14·5	(11·9–18·2)	19·6	(16·8–24·1)	34·2	(28·7–42·3)
Malaria	349	0·08	0·92	0·12	12·2	(9·1–13·9)	16·2	(13·4–19·6)	28·4	(22·5–33·5)
CNS Infections	216	0·06	0·85	0·26	10·0	(7·6–13·0)	11·0	(8·6–14·3)	21·0	(16·1–27·3)
Measles	161	0·04	0·89	0·08	5·1	(3·6–7·4)	10·2	(7·9–13·3)	15·3	(11·4–20·7)
Other Infectious Diseases	458	0·12	0·91	0·14	17·3	(14·3–21·1)	20·5	(17·7–24·7)	37·8	(23·5–47·3)
**Infectious Diseases Sub-Total**	**2246**	**0**·**58**	**0**·**88**	**0**·**13**	**84**·**2**		**111**·**7**		**196**·**0**	
Non Communicable Diseases/Other	792	0·21	0·82	0·20	37·4	(32·7–41·8)	33·6	(29·2–38·0)	71·0	(61·9–79·8)
Injuries	814	0·22	0·84	0·20	45·0	(40·1–49·70)	29·3	(25·0–33·4)	74·3	(65·1–83·1)
**Non-Infectious Disease Sub-Total**	**1606**	**0**·**42**	**0**·**83**	**0**·**20**	**82**·**4**		**62**·**9**		**145**·**3**	
**Total**	**3852**	**1**·**00**	**0**·**86**	**0**·**16**	**166**·**8**		**174**·**7**		**341**·**5**	

Footnote: MDS proportions are weighted according to Sample Registration System sampling fractions.

**Table 2:** Presence of Symptoms by Cause of Death (%).

Cause of Death	Number of Deaths	Symptom (%)					
		Fever	Diarrhea	Blood in Stool	Cough	Respiratory Distress	Fast Breathing
Diarrheal Diseases	675	56	82	20	22	19	56
Diarrheal Diseases w/o typhoid	565	49	93	20	18	16	56
Typhoid	110	98	25	18	40	31	55
Pneumonia	387	90	22	5	77	78	84
Malaria	349	98	15	14	34	29	75
CNS Infections	216	88	21	7	19	36	81
Measles	161	92	25	19	57	36	67
Other Infectious Diseases	458	82	17	17	38	32	66
**Total**	**2246**	**79**	**38**	**15**	**39**	**51**	**69**

The analysis of physician agreement demonstrates that physicians were highly likely to agree on both diarrhea and pneumonia as causes of death during the first review of the verbal autopsy (Table S3). The overall agreement was 94% for both causes. For diarrheal diseases inter-rater agreement (Cohen's kappa coefficient) was 0·79 (99% CI; 0·75–0·82) and for pneumonia it was 0·67 (99% CI; 0·61–0·72). Initial physician agreement exceeded 90% for boy and girl deaths for both diarrheal diseases and pneumonia.

**Figure 1:**
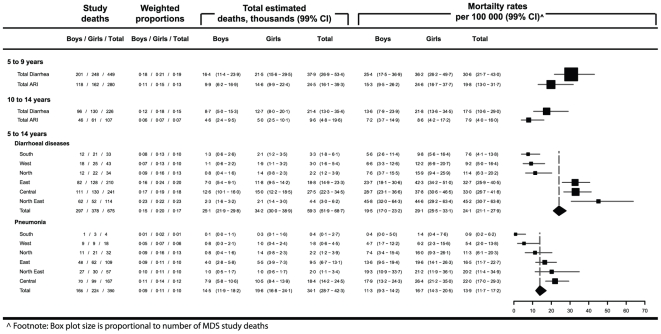
Estimated total deaths and mortality rates from diarrhea and pneumonia in children aged 5 to 14 years.

**Figure 2:**
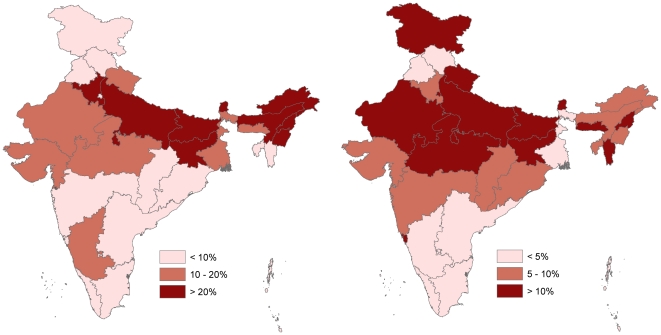
Diarrhea (left) and Pneumonia (right) Proportional Mortality.

**Figure 3:**
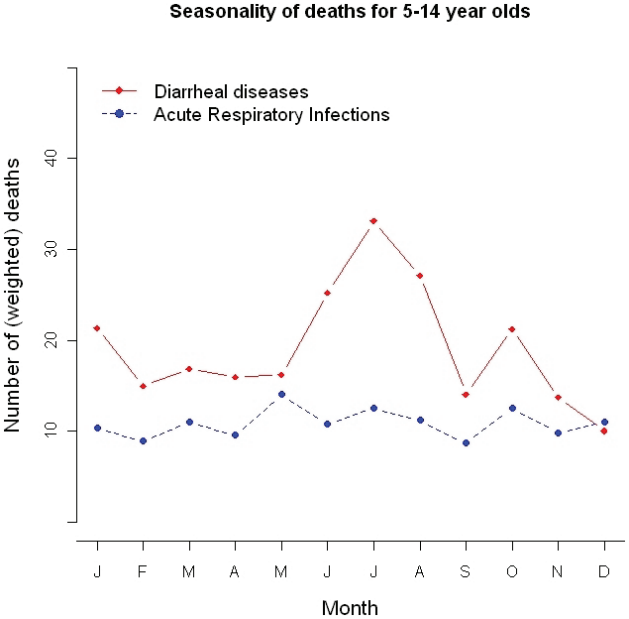
Seasonality of Diarrhea and Pneumonia Deaths.

We estimate there were approximately 59 300 (99% CI; 51 900 to 68 700) deaths due to diarrhea and 34 200 (99% CI; 28 700 to 42 300) deaths due to pneumonia in children aged 5 to 14 years in 2005. These correspond to mortality rates of 24·1 (99% CI; 21·1 to 27·9) and 13·9 (99% CI; 11·7 to 17·2) per 100 000 persons, respectively (Figure 1). Proportional mortality from both diarrhea and pneumonia were highest in the north of the country (Figure 2). Diarrheal deaths peaked in the period between June and August (Figure 3). There were significant differences in diarrhea mortality rates by region and gender. Girls had an approximately 50% higher mortality rate due to diarrhea than boys (43% greater in ages 5 to 9 years and 61% greater in ages 10 to 14 years). These differences result in approximately 35% more annual diarrheal deaths among girls (n = 34 200) compared to boys (n = 25 100). There was important regional variation; the diarrhea mortality rate was higher than 25 deaths per 100 000 children aged 5 to 14 years in the northeast, east, and central regions and lower than 12 in the north, west, and south regions. At ages 5 to 14 years, the mortality rate from pneumonia was approximately 50% higher in girls compared to boys, and in the 5 to 9 year old age group it was more than 60% higher in girls compared to boys. Similar to diarrhea mortality, the highest pneumonia mortality was seen in the east, central, and north east regions and the pneumonia mortality rate in the Central region (22·0 per 100 000 (99% CI; 17·0 to 29·3) was nearly 25 times higher than in the South (0·9 per 100 000 (99% CI; 0·2 to 6·2).

## Discussion

This is the first study to directly estimate causes of death in children aged 5 to 14 years in India. Infectious diseases cause over 196 000 annual deaths (approximately 60% of all deaths) in this age group. Diarrhea, pneumonia, and malaria are the three main infectious causes of death. We estimate that there were approximately 59 000 deaths due to diarrhea in 2005 (19% and 15% of all deaths in the 5 to 9 year and 10 to 14 year categories respectively) and 34 000 deaths due to pneumonia (13% of 5–9 years old deaths and 7% of 10 to 14 year old deaths).

One of the most important findings of our study is the gender difference in mortality; more girls than boys that died from not only infectious diseases in general but also each of the specific infectious diseases studied. For both diarrhea and pneumonia, mortality rates for girls were nearly 50% higher than boys. Increased mortality rates among Indian girls has previously been shown for children at ages 1 to 59 months [2] and these results show that excess mortality due to infectious diseases among girls extends into older childhood as well. This apparent gender bias may be enacted via parents being less likely to immunize [12], [13], seek medical attention [14], and/or being less likely to use appropriate antibiotic therapy for their sick female children [14]. The gender difference in infectious disease related mortality suggests that there are significant numbers of potential lives saved if mortality rates in girls could be reduced to those already seen in boys. As one example, if the national diarrhea mortality rate observed among children 5 to 14 years were as low as that observed among boys in the south (7·6 per 100,000 people, 99% CI; 4·1 to 13·8), there would be approximately 40 000 less diarrheal deaths nationally in this age group, a reduction of approximately 70%.

A review of data from the Registrar General of India's Survey of Causes of Death (SCD) [15] suggests that diarrhea mortality in older children has been high in India for some years. The SCD was conducted in a sample of villages from selected rural Primary Health Centres throughout India's major states. Table S4 shows the trends in mortality from diarrhea (diarrhea, cholera, food poisoning or dysentery) and pneumonia (pneumonia and whooping cough) from the SCD between 1991 and 1998. In the SCD, the annual proportion of deaths among children 5 to 14 years of age ranged from a low of 8·1% to a high of 13·1% for diarrhea and from a low of 6·4% to a high of 10·8% for pneumonia compared to 18% and 10% respectively in our study. While using a different methodology from our study, the SCD estimates support our findings that diarrhea is a major cause of death even among children older than 5 years of age. It is also worthwhile to examine other sources of information on causes of death in urban children aged 5 to 14 years. The Medically Certified Causes of Death (MCCD) [16] collects data in certain hospitals, generally in urban areas which are selected by the Chief Registrar of Births and Deaths. In 2004 the MCCD estimated that 2·5% of urban deaths in this age group were caused by diarrhea and 8·1% by pneumonia.

A recent systematic review examined published studies to estimate diarrhea morbidity among children aged 5 to 14 years and found that the median annual incidence rate was 67·5 episodes (IQR 15·4–91·7) per 100 person-years in the WHO South East Asia Region [17]. The same review included six studies of diarrhea mortality in older children, adolescents, and adults; however, only two of these studies included children aged 5 to 14 years [17]. One of these studies, conducted in Indonesia, found that diarrheal diseases were the cause of 22·2% of all deaths in children aged 5 to 14 years [18].

A larger study that used physician coded verbal autopsies in Senegal found that 12·5% of all deaths in the 5 to 14 year old age group were caused by diarrhea [19]. Though limited in number and in scope, the published literature is consistent in its findings. Our study now provides the most reliable evidence to date for India, based on the largest number of deaths, and the first from South Asia, to corroborate that diarrhea is a major cause of death in children older than five years.

For diarrheal diseases, we used the commonly used definition of Intestinal Infectious Diseases from the ICD-10 (codes A00 to A09). Of all diarrheal deaths studied, approximately 80% were physician coded as A09 (infectious gastroenteritis and colitis) and 16% were coded as A01 (typhoid and paratyphoid fevers) (Table S2). In young children, typhoid is less likely to be associated with diarrhea symptoms and more likely to include fever, constitutional symptoms, gastrointestinal disturbances and hepatosplenomegaly [20], [21]. The different clinical presentations of deaths coded as gastroenteritis and typhoid fevers is evident in Table 2. Overall, 16·3% (110/675) of the diarrheal deaths were coded as typhoid/paratyphoid, corresponding to approximately 9000 annual diarrheal deaths due to typhoid and paratyphoid fever in children aged 5 to 14 years in India.

Diarrhea, pneumonia, central nervous system infections, and measles represent nearly 130 000 annual deaths in this age group and are all, to a degree, vaccine preventable diseases. While the microbiologic etiology of severe diarrheal disease in older children remains incompletely understood, both rotavirus and *Salmonella typhi* are likely players and both are preventable by safe and effective vaccines. *Streptococcus pneumoniae* and *Haemophilus influenza* type B are well recognized blood, lung, and central nervous system pathogens and these two organisms have been estimated to cause over 200 000 deaths in all ages in India annually [22], [23]. Infections from both of these organisms, in addition to *Neisseria meningitides*, another major bacterial cause of central nervous system infections, are largely preventable through the use of existing vaccines. Measles, has been effectively eliminated in much of the world through vaccination. The availability of effective preventive tools and the fact that control of infectious diseases (including diarrhea, pneumonia, central nervous system infections, and measles) have not been made a priority in this age group suggest that there is significant potential to reduce mortality from these causes among older children in India.

There are several limitations to this study, the major one being that verbal autopsy based studies may be biased by misclassification of causes of death. An analysis of over 27 000 childhood deaths in the MDS did find that physicians were more likely to agree on the categorical cause of death in children aged 5 to 14 years than in any other age category [24]. Additionally, the symptoms based tabulation (table 2) is very much consistent with what would be expected given the clinical presentation of the various conditions. However, this does not necessarily validate that the cause of death agreed upon by the physicians is in fact correct. Validation studies comparing verbal autopsy classification of major causes of death to known hospital diagnoses or death certificates as the gold standard have been conducted for children under 5, adults, and maternal deaths [25]–[30], however, it is likely that there are differences between hospital deaths and deaths that occur at home. To our knowledge, no such validation study has been performed specifically on deaths between the ages of 5 to 14 years. Multiple verbal autopsy validation studies in children show that the sensitivity and specificity of verbal autopsy diagnosed diarrhea and pneumonia versus medically certified cause of death is in general reasonable. For diarrhea, studies from the Philippines [31], Namibia [32], Bangladesh [33], South Africa [27], Uganda [33], and Nicaragua [33], [34] show sensitivities ranging from 51% to 88% and specificities ranging from 54% to 100%. For pneumonia, the same studies [27], [31]–[34] show sensitivities ranging from 58% to 91% and specificities from 37% to 91%. While there is an inherent possibility of misclassification in any individual death, for major causes of death verbal autopsy does provide estimations of overall causes of death within the population sufficient to make actionable policy recommendations.

Another limitation is that we were unable to determine the contribution of underlying medical co-morbidities, such as caloric or protein malnutrition, micronutrient deficiencies, immune deficiencies, and others, that may have contributed to the deaths. We are also unable to determine role that poor access to health services, most specifically oral or intravenous rehydration for diarrheal disease and antibiotic therapy and respiratory supportive care for pneumonia, may have played in the deaths of these children. However, the fact that over 85% of both the diarrheal and pneumonia deaths in this study occurred in rural areas and only 12% of diarrhea deaths and 10% of pneumonia deaths occurred in health centres suggests that the majority of care for these children, if any, was provided locally and likely by those lacking comprehensive health training.

### Conclusions

The starting point for reducing mortality must be a thorough understanding of the causes, burden, and distribution of deaths. In this study, we present the first direct national estimates of causes of death among Indian children aged 5 to 14 years. Approximately 60% of deaths in this age group are due to infectious diseases and about 59 000 and 34 000 of these are due to diarrhea and pneumonia respectively. Significant variation in infectious disease mortality, including from diarrhea and pneumonia, in older children by gender and by region is noted: girls and children in the Central, East, and Northeast regions of the country are at a much higher risk.

The majority of global childhood mortality research focuses on children under the age of 5 years and there are very limited data on cause specific mortality in older children. As a result, the burden of mortality, and in particular the burden from diarrheal diseases, in older children may have been largely unrecognized in many countries, including India. Further research into the etiology, prevention, and management of infectious diseases, including severe diarrhea and pneumonia, in older children, combined with a new understanding of the burden and distribution of disease among older children, holds the potential to significantly reduce overall mortality, as well as minimize gender and geographic inequities.

## Supporting Information

Table S1 Cause of death classification for children aged 5 to 14 years.
(DOC)

Table S2 MDS Diarrheal and Pneumonia Deaths in Children Aged 5 to 14 Years.
(DOC)

Table S3 Physician Agreement and Kappa Analysis of Diarrheal and Pneumonia Deaths.
(DOC)

Table S4 Trends in Diarrhea and Pneumonia Proportional Mortality from India Survey of Causes of Death.
(DOC)

## References

[1] Black RE, Morris SS, Bryce J (2003). Where and why are 10 million children dying every year?. Lancet.

[2] Bassani DG, Kumar R, Awasthi S, Morris SK, Million Death Study Collaborators (2010). Causes of neonatal and child mortality in India: A nationally representative mortality survey.. Lancet.

[3] Lopez AD, Mathers CD, Ezzati M, Jamison DT, Murray CJ (2006). Global burden of disease and risk factors.

[4] Jha P, Gajalakshmi V, Gupta PC, Kumar R, Mony P (2006). Prospective study of one million deaths in India: Rationale, design, and validation results.. PLoS Med.

[5] Registrar General of India (2004).

[6] Centre for Global Health Research (2009). Million death study..

[7] World Health Organization, editor (2007). International statistical classification of diseases and related health problems, 10th revision.

[8] United Nations, Department of Economic and Social Affairs (DESA). World population prospects, the 2008 revision.

[9] Jha P, Jacob B, Gajalakshmi V, Gupta PC, Dhingra N (2008). A nationally representative case-control study of smoking and death in India.. N Engl J Med.

[10] StataCorp (2007). Stata statistical software: Release 10.

[11] Dhingra N, Jha P, Sharma VP, Cohen AA, Jotkar RM (2010). Adult and child malaria mortality in India: A nationally representative mortality survey.. Lancet.

[12] Borooah VK (2004). Gender bias among children in India in their diet and immunisation against disease.. Soc Sci Med.

[13] Pande RP, Yazbeck AS (2003). What's in a country average? wealth, gender, and regional inequalities in immunization in India.. Soc Sci Med.

[14] Srivastava SP, Nayak NP (1995). The disadvantaged girl child in Bihar: Study of health care practices and selected nutritional indices.. Indian Pediatr.

[15] Registrar General of India (1998). Survey of causes of death (rural): Annual report.

[16] Registrar General of India (2009). Report on medical certification of causes of death 2004.

[17] Walker CL, Black RE (2010). Diarrhoea morbidity and mortality in older children, adolescents, and adults.. Epidemiol Infect.

[18] Anwar Z, Djamil H, Pardede N, Ismail R (1987). The pattern of the causes of death in children in rural swampy area of south sumatra, Indonesia.. Paediatr Indones.

[19] Etard JF, Le Hesran JY, Diallo A, Diallo JP, Ndiaye JL (2004). Childhood mortality and probable causes of death using verbal autopsy in Niakhar, Senegal, 1989–2000.. Int J Epidemiol.

[20] Bhan MK, Bahl R, Bhatnagar S (2005). Typhoid and paratyphoid fever.. Lancet.

[21] Bhutta ZA (2006). Current concepts in the diagnosis and treatment of typhoid fever.. BMJ.

[22] Watt JP, Wolfson LJ, O'Brien KL, Henkle E, Deloria-Knoll M (2009). Burden of disease caused by *Haemophilus influenzae* type b in children younger than 5 years: Global estimates.. Lancet.

[23] O'Brien KL, Wolfson LJ, Watt JP, Henkle E, Deloria-Knoll M (2009). Burden of disease caused by *Streptococcus pneumoniae* in children younger than 5 years: Global estimates.. Lancet.

[24] Morris SK, Bassani DG, Kumar R, Awasthi S, Paul VK (2010). Factors associated with physician agreement on verbal autopsy of over 27000 childhood deaths in India.. PLoS One.

[25] Gajalakshmi V, Peto R (2004). Verbal autopsy of 80,000 adult deaths in Tamilnadu, south India.. BMC Public Health.

[26] Gajalakshmi V, Peto R, Kanaka S, Balasubramanian S (2002). Verbal autopsy of 48 000 adult deaths attributable to medical causes in Chennai (formerly Madras), india.. BMC Public Health.

[27] Kahn K, Tollman SM, Garenne M, Gear JS (2000). Validation and application of verbal autopsies in a rural area of South Africa.. Trop Med Int Health.

[28] Setel PW, Whiting DR, Hemed Y, Chandramohan D, Wolfson LJ (2006). Validity of verbal autopsy procedures for determining cause of death in Tanzania.. Trop Med Int Health.

[29] Chandramohan D, Maude GH, Rodrigues LC, Hayes RJ (1998). Verbal autopsies for adult deaths: Their development and validation in a multicentre study.. Trop Med Int Health.

[30] Chandramohan D, Rodrigues LC, Maude GH, Hayes RJ (1998). The validity of verbal autopsies for assessing the causes of institutional maternal death.. Stud Fam Plann.

[31] Kalter HD, Gray RH, Black RE, Gultiano SA (1990). Validation of postmortem interviews to ascertain selected causes of death in children.. Int J Epidemiol.

[32] Mobley CC, Boerma JT, Titus S, Lohrke B, Shangula K (1996). Validation study of a verbal autopsy method for causes of childhood mortality in Namibia.. J Trop Pediatr.

[33] Anker M, Black RE, Coldham C, Kalter HD, Quigley MA (1999). A standard verbal autopsy method for investigating causes of death in infants and children.. http://www.who.int/csr/resources/publications/surveillance/whocdscsrisr994.pdf.

[34] Coldham C, Ross D, Quigley M, Segura Z, Chandramohan D (2000). Prospective validation of a standardized questionnaire for estimating childhood mortality and morbidity due to pneumonia and diarrhoea.. Trop Med Int Health.

