# Correction: Diarrhea, Pneumonia, and Infectious Disease Mortality in Children Aged 5 to 14 Years in India

**DOI:** 10.1371/annotation/65b7652d-fad0-4595-90b1-77f9685cf25f

**Published:** 2013-11-15

**Authors:** Shaun K. Morris, Diego G. Bassani, Shally Awasthi, Rajesh Kumar, Anita Shet, Wilson Suraweera, Prabhat Jha, for the MDS Collaborators

A funding organization was incorrectly omitted from the Funding Statement. The following sentence should be read with the Funding Statement: “This study was prepared with partial support from the Bill and Melinda Gates Foundation through the Disease Control Priorities Network at the University of Washington.” 

