# Supplementary material for: Diarrhea, Pneumonia, and Infectious Disease Mortality in Children Aged 5 to 14 Years in India
Source: PLoS One. 2011 May 24;6(5):e20119. doi: 10.1371/journal.pone.0020119 (PMC3101242; doi:10.1371/journal.pone.0020119)
Supplement: Table S1 — Cause of death classification for children aged 5 to 14 years. (DOC) [file pone.0020119.s001.doc]

Table S1: Cause of death classification for children aged 5 to 14 years.

| **5 to 14 years category** | **ICD-10 codes** |
| --- | --- |
|  |  |
| Pneumonia | A37, H65-H68, H70, H71, J00-J06, J09-J18, J20-J22, J32, J36, J85, J86, P23, U04 |
| Diarrhoeal diseases | A00-A09 |
| Measles | B01, B05 |
| Other noncommunicable diseases | C00-C26, C30-C34, C37-C41, C43-C58, C60-C85, C88, C90-C97, D01-D07, D09-D48, D55-D77, D80-D84, D86, D89,  E03-E07, E10-E16, E20-E32, E34, E35, E65-E68, E70-E80, E83-E90, F00-F07, F09-F25, F28-F34, F38-F45, F48, F50-  F55, F59-F66, F68-F73, F78-F84, F88-F95, F98, F99, G10-G13, G20-G26, G30-G32, G35-G37, G40, G41, G43-G47,  G50-G64, G70-G73, G80-83, G90-G99, H00-H06, H11, H13, H15-H22, H25-H28, H30-H36, H40, H42, H43-H55, H57-  H59, H61, H62, H69, H72-H75, H80-H83, H90-H95, I00-I02, I05-I13, I15, I20-I28, I3, I34-I38, I42-I52, I60-I74, I77-I89,  I95, I97-I99, J30, J31, J33-J35, J37-J47, J60, J64, J66-J70, J80-J82, J84, J90-J96, J98, J99, K00-K03, K06-K14, K20-  K23, K25-K31, K35-K38, K40-K46, K50-K52, K55-K60, K62, K63, K70-K77, K80, K82, K83, K85-K87, K90-K93, L05,  L10-L14, L20-L30, L40-L45, L50-L60, L62-L68, L70-L75, L80-L95, L97-L99, M02, M03, M05-M25, M30-M36, M40-M43,  M45-M51, M53, M54, M61-M63, M65-M68, M70-M73, M75-M77, M79-M85, M87-M96, M99, N00-N08, N11-N23, N25-  N29, N31-N33, N35-N37, N39, N40, N42-N48, N50, N51, N60, N62-N64, N75-N77, N80-N99, P04, P08, P51, P53-P60,  P70-P72, P74-P76, P78, P80, P81, P83, P92-P94, R00, R01, R03-R05, R06, R11-R23, R26, R27, R29-R36, R39-R49, |
| Injuries | S00-S99, T00-T71, T73-T75, T78-T98, V01-V06, V09-V99, W00-W46, W49-W60, W64-W70, W73-W81, W83-W94, W99,  X00-X06, X08-X52, X57-X99, Y00-Y36, Y40-Y66, Y69-Y91, Y96-Y98 |
| Malaria | B50-B54 |
| Meningitis/encephalitis | A39, A81-A89, G00-G09 |
| Nutritional diseases | D50-D53, E00-E02, E40-E46, E50-E56, E59-E61, E63, E64, X53, X54 |
| Acute bacterial sepsis & severe infections | A20-A28, A32, A38, A40-A44, A46, A48, A49, A68-A70, A74, A75, A77-A79, B95, B96, H10, H60, I30, I32, I33, I39-I41,  K65, K67, K81, L00-L04, L08, M00, M01, M60, M86, N10, N30, N34, N41, N49, N61, P36, P38 |
| Other infectious and parasitic diseases | A30, A31, A36, A50-A60, A63-A67, A71, A90-A96, A98, A99, B00, B02-B04, B06-B09, B15-B19, B25-B27, B30, B33-B49,  B55--B60, B64-B83, B85-B89, B92, B94, B97, B99, K02, K04, K05, K61, N70-N74, P35, P37, P39, U00, Y95. |
| Other causes: |  |
| *Birth asphyxia and birth*  *trauma* | P00, P02, P03. P10-P15, P20, P21, P24, P29, P50, P90, P91 |
| *Congenital anomalies* | Q00-Q07, Q10-18, Q20-Q28, Q30-Q45, Q50-56, Q60-Q87, Q89-Q93, Q95, Q96-Q99 |
| *Fever of unknown origin* | R50 |
| *HIV/AIDS* | B20-B24, R75 |
| *Ill defined or cause*  *unknown* | P96, R02, R07, R09, R10, R25, R51-R54, R57-R58, R60-R62, R64, R68, R69, R78, R79, R83, R89, R92-R96, R98, R99 |
| *Poliomyelitis* | A80, B91 |
| *Prematurity &*  *intrauterine growth*  *restriction* | P01, P05, P07, P22, P25-P28, P52, P61, P77 |
| *Tetanus* | A33-A35 |
| *Tuberculosis* | A15-A19, B90, J65 |
