# Supplementary material for: Diarrhea, Pneumonia, and Infectious Disease Mortality in Children Aged 5 to 14 Years in India
Source: PLoS One. 2011 May 24;6(5):e20119. doi: 10.1371/journal.pone.0020119 (PMC3101242; doi:10.1371/journal.pone.0020119)
Supplement: Table S2 — MDS Diarrheal and Pneumonia Deaths in Children Aged 5 to 14 Years. (DOC) [file pone.0020119.s002.doc]

Table S2: MDS Diarrheal and Pneumonia Deaths in Children Aged 5 to 14 Years

|  | **ICD10 Code** | **Name** | **MDS #** |
| --- | --- | --- | --- |
|  |  |  |  |
| **Diarrhea** | A09 | Infectious gastroenteritis and colitis, | 547 |
|  | A01 | Typhoid and paratyphoid fevers | 110 |
|  | A00 | Cholera | 10 |
|  | A05 | Other bacterial foodborne intoxications | 4 |
|  | A03 | Shigellosis | 2 |
|  | A06 | Viral and other specified intestinal in | 1 |
|  | A08 | Amoebiasis | 1 |
|  | **Total** |  | **675** |
|  |  |  |  |
| **Pneumonia** | J18 | Pneumonia, organism unspecified | 206 |
|  | J22 | Unspecified acute lower respiratory inf | 140 |
|  | J11 | Influenza, virus not identified | 17 |
|  | J16 | Pneumonia due to other infectious organ | 7 |
|  | J15 | Bacterial pneumonia, not elsewhere clas | 5 |
|  | J20 | Acute bronchitis | 4 |
|  | A37 | Whooping cough | 3 |
|  | J03 | Acute tonsillitis | 2 |
|  | J06 | Acute upper respiratory infections of m | 2 |
|  | H66 | Abscess of lung and mediastinum | 1 |
|  | J05 | Acute bronchiolitis | 1 |
|  | J21 | Acute obstructive laryngitis [croup] an | 1 |
|  | J85 | Suppurative and unspecified otitis medi | 1 |
|  | **Total** |  | **390** |
