# Supplementary material for: Diarrhea, Pneumonia, and Infectious Disease Mortality in Children Aged 5 to 14 Years in India
Source: PLoS One. 2011 May 24;6(5):e20119. doi: 10.1371/journal.pone.0020119 (PMC3101242; doi:10.1371/journal.pone.0020119)
Supplement: Table S3 — Physician Agreement and Kappa Analysis of Diarrheal and Pneumonia Deaths. (DOC) [file pone.0020119.s003.doc]

Table S3: Physician Agreement and Kappa Analysis of Diarrheal and Pneumonia Deaths

|  |  |  | **Study**  **Deaths** | **Agreement (%)** | **Kappa** | **99% CI** |
| --- | --- | --- | --- | --- | --- | --- |
|  |  |  |  |  |  |  |
| **Diarrheal Diseases** | 5 to 9 years | Boys | 201 | 94·2 | 0·80 | (0·74-0·86) |
|  |  | Girls | 248 | 92·9 | 0·79 | (0·73-0·85) |
|  |  | Total | 449 | 93·6 | 0·79 | (0·75-0·84) |
|  |  |  |  |  |  |  |
|  | 10 to 14 years | Boys | 96 | 96·4 | 0·84 | (0·76-0·91) |
|  |  | Girls | 130 | 92·3 | 0·72 | (0·63-0·81) |
|  |  | Total | 226 | 94·4 | 0·77 | (0·71-0·83) |
|  |  |  |  |  |  |  |
|  | 5 to 14 years | Boys | 297 | 95·1 | 0·81 | (0·77-0·86) |
|  |  | Girls | 378 | 92·7 | 0·77 | (0·72-0·81) |
|  |  | Total | 675 | 93·9 | 0·79 | (0·75-0·82) |
|  |  |  |  |  |  |  |
|  |  |  |  |  |  |  |
| **Pneumonia** | 5 to 9 years | Boys | 118 | 94·2 | 0·70 | (0·60-0·79) |
|  |  | Girls | 162 | 91·7 | 0·65 | (0·56-0·74) |
|  |  | Total | 280 | 92·9 | 0·67 | (0·61-0·73) |
|  |  |  |  |  |  |  |
|  | 10 to 14 years | Boys | 46 | 97·2 | 0·70 | (0·54-0·85) |
|  |  | Girls | 61 | 94·1 | 0·60 | (0·46-0·74) |
|  |  | Total | 107 | 95·7 | 0·64 | (0·54-0·75) |
|  |  |  |  |  |  |  |
|  | 5 to 14 years | Boys | 164 | 95·4 | 0·70 | (0·62-0·78) |
|  |  | Girls | 223 | 92·7 | 0·64 | (0·57-0·71) |
|  |  | Total | 387 | 94·0 | 0·67 | (0·61-0·72) |
