# Supplementary material for: Diarrhea, Pneumonia, and Infectious Disease Mortality in Children Aged 5 to 14 Years in India
Source: PLoS One. 2011 May 24;6(5):e20119. doi: 10.1371/journal.pone.0020119 (PMC3101242; doi:10.1371/journal.pone.0020119)
Supplement: Table S4 — Trends in Diarrhea and Pneumonia Proportional Mortality from India Survey of Causes of Death. (DOC) [file pone.0020119.s004.doc]

Table S4: Trends in Diarrhea and Pneumonia Proportional Mortality from India Survey of Causes of Death

|  |  |  |  | **1991** | **1992** | **1993** | **1994** | **1995** | **1996** | **1997** | **1998** |
| --- | --- | --- | --- | --- | --- | --- | --- | --- | --- | --- | --- |
|  |  |  |  |  |  |  |  |  |  |  |  |
| **Diarrheal Diseases** | | **5-9 years** |  | 13·5 | 10·9 | 13·2 | 13·0 | 8·2 | 7·8 | 8·8 | 6·9 |
|  |  | **10-14 years** | | 9·2 | 10·8 | 8·1 | 11·9 | 8·3 | 6·9 | 8·8 | 7·0 |
|  |  | **5-14 years** | | 12·6 | 11·7 | 11·8 | 13·1 | 9·2 | 8·6 | 9·5 | 8·1 |
|  |  |  |  |  |  |  |  |  |  |  |  |
|  |  |  |  |  |  |  |  |  |  |  |  |
| **Pneumonia** | | **5-9 years** |  | 12·2 | 11·7 | 13·4 | 10·2 | 10·4 | 10·6 | 9·7 | 8·9 |
|  |  | **10-14 years** | | 8·8 | 4·6 | 4·6 | 4·0 | 5·3 | 3·6 | 4·0 | 3·6 |
|  |  | **5-14 years** | | 10·8 | 8·8 | 9·9 | 7·4 | 8·1 | 7·5 | 7·2 | 6·4 |
